# On the Use of the Ethereal Solution of Gun Cotton as an External Application in Erysipelas

**Published:** 1850-01

**Authors:** J. W. Freer

**Affiliations:** The Grove, Cook Co., Ills.


### ARTICLE III.

*On the Use of the Etherial Solution of Gun Cotton as an External Application in Erysipelas.*—By J. W. Freer, M.D., of The Grove, Cook Co., Ills.

Having made use of the adhesive liquid plaster as an external application in erysipelas, with the most gratifying success, I thought it not improper to make known the results.

An epidemic of the above named disease prevailed in our vicinity last Spring, and annoyed me not a little, to find external remedies to alleviate the smarting, burning pain of the inflammation, and to prevent it from spreading over the surface. Reasoning from the fact that such inflammations are usually superficial, involving principally the capillary system of the cuticle and subcutaneous tissues, it seemed reasonable to suppose that any substance which would, after application, contract, thereby expelling the superabundance of blood from the part, would of course lessen the pain and irritation.—After experimenting, my anticipations were fully realized.

The first trial was upon a boy about 10 years of age. The inflammation commenced at the nose, and continued to travel until it had involved the whole of the face, scalp, neck, and finally passed down the back—ultimately uniting in front.—The pain and irritation resulting from the inflammation, added to the constitutional symptoms, made the case appear quite hopeless. At this period the solution was applied by means of a feather over the whole of the recently involved surface, and immediate relief was given. The inflammatory redness disappeared, and a firm coating was given which entirely protected the parts from the air, and the contact of clothing. The patient soon began to recover rapidly.

Afterwards I tried it in many instances with like success, with this addition, that no case afterwards traveled beyond its limits at the time of application. I do not presume to say that the spreading was prevented by it, for the inflammation might not have gone beyond those limits without its agency. Since then I have had occasion to use it in other affection, the most important of which are burns. It forms a firm coating, excluding the air, and almost instantaneously relieving the pain. In common inflammation, from whatever cause arising, its application seems to promote a termination by resolution, acting upon the same principle as in erysipelas, that is by squeezing, as it contracts, the fluids from the parts, thereby reducing the morbid action.

